# The Absence of a Mature Cell Wall Sacculus in Stable *Listeria monocytogenes* L-Form Cells Is Independent of Peptidoglycan Synthesis

**DOI:** 10.1371/journal.pone.0154925

**Published:** 2016-05-05

**Authors:** Patrick Studer, Marina Borisova, Alexander Schneider, Juan A. Ayala, Christoph Mayer, Markus Schuppler, Martin J. Loessner, Yves Briers

**Affiliations:** 1 Institute for Food, Nutrition and Health, ETH Zurich, Zurich, Switzerland; 2 Department of Microbiology/Biotechnology, University of Tuebingen, Tuebingen, Germany; 3 Centro de Biología Molecular Severo Ochoa, Universidad Autónoma de Madrid, Madrid, Spain; 4 Department of Applied Biosciences, Ghent University, Ghent, Belgium; Institut Pasteur Paris, FRANCE

## Abstract

L-forms are cell wall-deficient variants of otherwise walled bacteria that maintain the ability to survive and proliferate in absence of the surrounding peptidoglycan sacculus. While transient or unstable L-forms can revert to the walled state and may still rely on residual peptidoglycan synthesis for multiplication, stable L-forms cannot revert to the walled form and are believed to propagate in the complete absence of peptidoglycan. L-forms are increasingly studied as a fundamental biological model system for cell wall synthesis. Here, we show that a stable L-form of the intracellular pathogen *Listeria monocytogenes* features a surprisingly intact peptidoglycan synthesis pathway including glycosyl transfer, in spite of the accumulation of multiple mutations during prolonged passage in the cell wall-deficient state. Microscopic and biochemical analysis revealed the presence of peptidoglycan precursors and functional glycosyl transferases, resulting in the formation of peptidoglycan polymers but without the synthesis of a mature cell wall sacculus. In conclusion, we found that stable, non-reverting L-forms, which do not require active PG synthesis for proliferation, may still continue to produce aberrant peptidoglycan.

## Introduction

L-forms are cell wall-deficient bacterial cells that usually possess a cell wall, but can survive and multiply in the absence of this structure. The wall-deficient state can be temporal (unstable/transient L-forms) or permanent (stable L-forms). For long, it has been believed that L-forms do not produce peptidoglycan (PG), or at least do not require active PG synthesis [1]. However, a more nuanced view on the presence and role of PG in L-forms has gained acceptance in the last decade. It has now been shown that some L-form strains require PG synthesis for multiplication [2–4], while other L-forms can propagate in the complete absence of PG synthesis [5–8].

With respect to the first group of L-forms, which still require PG synthesis, Joseleau-Petit and colleagues described an unstable *E*. *coli* L-form strain generated by exposure to the β-lactam antibiotic cefsulodin, a specific inhibitor of penicillin-binding proteins (PBPs) 1A and 1B, in rich hypertonic medium [2]. Genetic knockouts demonstrated the requirement of D-glutamate and diaminopimelate, specific precursor molecules for PG synthesis. Also MurA that catalyzes the first reaction in the synthesis of the muramic acid side chain is essential for L-form propagation. The use of specific inhibitors of PBP2 and PBP3 (septal PG synthesis) showed that both PBPs are required for L-form growth and multiplication. A residual amount of 7% PG in comparison to the normal cells was quantified with HPLC, with the average length of glycan chains one-third shorter in the L-form state. The residual PG in this type of *E*. *coli* L-forms was located with a fluorescently labelled PG binding protein and fluorescent D-amino acids on small buds that are formed during a budding-like division process and at constriction sites [3, 4], which is consistent with a role of septal PG synthesis for multiplication. During reversion to the walled state, the unstable *E*. *coli* L-form cells are quickly covered by a new PG layer, which is extending from those small buds. MreB has an essential function during this reversion process. This actin homologue is involved in the spatiotemporal regulation of PG synthesis of rod-shaped bacteria. During reversion of L-forms, MreB locates to inwardly curved regions of the cell membrane and targets PG synthesis to those regions, thereby governing the establishment of the rod shape [4].

A completely different type of L-form cells are those that can grow in absence of any PG synthesis. They have been generated by deleting or suppressing genes essential for PG synthesis [6], by prolonged passage in the presence of penicillin [5], or by inhibiting an early step of PG synthesis using specific antibiotics [7]. The proliferation process of certain *B*. *subtilis* L-forms from this group is independent of FtsZ and based on membrane blebbing and scission, driven by increased membrane synthesis [6, 9]. Importantly, branched-chain fatty acid synthesis is essential to achieve the necessary membrane fluidity for membrane scission [10]. The dispensability of the common cell wall synthesis machinery together with the observation that purely biophysical processes enable proliferation of these L-forms [9] support the hypothesis that L-forms fall back on a primitive proliferation mechanism that was used by primitive bacteria before the evolution of the cell wall [11, 12]. Interestingly, even L-forms that grow in absence of essential cell wall synthesis genes can again synthesize a complete cell wall sacculus when the missing genes are reintroduced, conclusively demonstrating that no preexisting template is needed for *de novo* synthesis of a cell wall [13].

The inability of stable L-forms to regenerate a cell wall is hypothesized to be related to genetic changes in the PG synthesis pathway [14]. Such mutations were identified in the partial sequence of a stable protoplast-type *E*. *coli* L-form that was generated by prolonged passage in the presence of penicillin. This strain contained, amongst other genetic changes, a non-sense mutation in the lipid II synthesis gene *mraY*, which leads to an inactive, N-terminally truncated protein and thus prevents the synthesis of PG [15]. However, it cannot be differentiated if this mutation is the primary cause of stabilization, or a tolerated mutation that arose during the long period (approximately 40 years) of subcultivation after stabilization.

Since PG and the muropeptides generated during cell-wall turnover are known to be modulators of the host immune response and are important determinants for virulence of pathogenic bacteria, L-forms are proposed to have a selective advantage over walled bacteria [16–19]. This is especially the case for stable L-forms, which no longer have the capability to produce a mature cell wall sacculus.

In this study, such a stable L-form was generated from the intracellular pathogen *Listeria monocytogenes* by prolonged passage in presence of penicillin G, and used for the study of the cell wall synthesis pathway in stable L-forms. Genome analysis of this L-form strain revealed many mutations, but did not indicate which part of the cell wall synthesis pathway is still intact. Therefore, sequential steps of cell wall synthesis were analyzed in detail. Although this L-form strain is able to grow in absence of any cell wall synthesis, a partially active cell wall synthesis pathway with PG precursor production and PG glycosyl transfer reaction was found. Thus, from the absence of an intact mature cell wall sacculus, it cannot be concluded that PG synthesis is completely defective, challenging the suggested selective advantage of L-forms over walled cells with regard to immune stimulation and recognition.

## Results

### Stable Listeria L-forms do not require residual PG synthesis for growth

**Fig 1:**
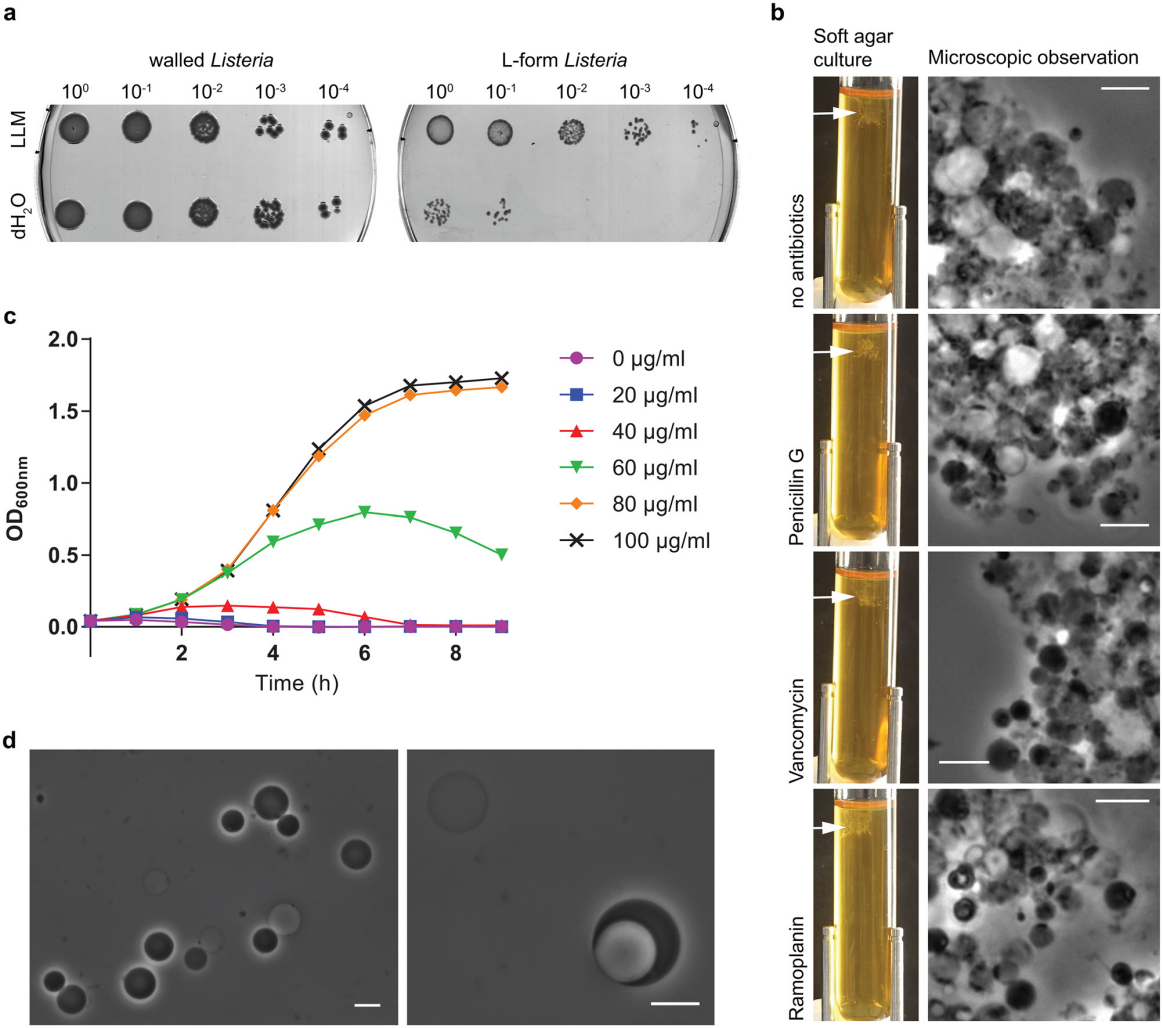
Cell wall synthesis is not essential for L-form growth. (a) L-forms are highly sensitive to osmotic differences. Walled or L-form cells were exposed to deionized water or *Listeria* L-form medium (LLM) as control. After 5 min exposure, a ten-fold dilution series was spotted on agar plates to enumerate the surviving bacteria. Whereas no difference between water and LLM treated samples could be observed for walled *Listeria*, more than 99% of the L-forms died when exposed to deionized water. (b) L-forms can grow in presence of various antibiotics that specifically inhibit transpeptidation (penicillin G; 50 μg/ml), glycosyl transfer (ramoplanin, 6.25 μg/ml) or both (vancomycin, 100 μg/ml). Arrows indicate growing colonies within soft agar tubes. Scale bar, 10 μm. (c) D-alanine dependency of the walled form of a mutant which is deficient in the alanine racemase and the D-amino acid aminotransferase gene. The legend shows the D-alanine concentration in the BHI medium, which only allows growth of walled bacteria. (d) L-forms derived from the D-alanine auxotroph *Listeria* strain grown in *Listeria* L-form medium. Scale bar, 10 μm.

By passaging a culture derived from walled *L*. *monocytogenes* EGDe in *Listeria* L-form medium (LLM) with high concentrations of penicillin G for more than two years, we generated a stable L-form strain that is unable to revert to the walled form. The spherical shape indicates the absence of a mature cell wall sacculus in L-forms. In addition, exposure of these L-forms to deionized water resulted in a 2–3 log reduction of the viable cell count, whereas exposure of walled *Listeria* to deionized water does not decrease the cell number, showing the increased sensitivity to changes in osmolarity as a consequence of the lack of a intact cell wall sacculus (Fig 1a). To investigate, whether this strain requires residual cell wall synthesis for proliferation, we inoculated a culture grown in absence of antibiotics into soft agar medium supplemented with either no antibiotics, penicillin G, vancomycin or ramoplanin. Penicillin G, which was also used for L-form induction, specifically inhibits transpeptidation. Ramoplanin in contrast specifically inhibits the glycosyl transferase reaction [20, 21], whereas vancomycin inhibits both, transpeptidation and glycosyl transfer [22]. The concentrations of the different antibiotics corresponded to 25x MIC as determined for walled *Listeria* cells in LLM. After three days of incubation, all tubes showed growth, which was both microscopically and macroscopically visible (Fig 1b), indicating that residual cell wall synthesis (glycosyl transferase activity and transpeptidation) is not required for multiplication in the L-form state.

To further support this observation, a genetically engineered strain derived from *L*. *monocytogenes* 10403S, in which an early step of the PG synthesis pathway is interrupted, was tested for its capability to grow as an L-form. This strain is deficient in the alanine racemase (*dal*) and the D-amino acid aminotransferase (*dat*) gene and therefore not capable of producing D-alanine [23], which is part of the stem peptide of the lipid II precursor molecules. However, when D-alanine is supplemented externally, the cells show a usual sigmoid growth curve in BHI medium (Fig 1c) and are rod-shaped (data not shown). The effect of externally added D-alanine is dose-dependent, and a complete lack of growth is observed with 0 and 20 μg/ml D-Ala. Following inoculation of the D-Ala auxotroph in *Listeria* L-form medium without D-Ala, L-forms were induced spontaneously (Fig 1d) and we found that the cells propagated well in the L-form state. Taking together, the lack of inhibition under high antibiotic concentrations and the ability of the D-Ala auxotroph to grow and multiply are consistent with the lack of need of peptidoglycan synthesis.

### Stable L-forms accumulate various mutations

Stable L-forms are proposed to contain mutations in the cell wall synthesis pathway, which prevent the reversion to the walled state [14]. To identify such potential mutations in the penicillin G induced stable L-form, the genome of this strain was sequenced. Alignment with the published *L*. *monocytogenes* EGDe reference (NCBI Reference Sequence: NC_003210), and in-house re-sequenced *L*. *monocytogenes* EGDe strains revealed 206 non-silent mutations, including the loss of the A118-like prophage normally integrated into *comK* [24] (S1 Table). Multiple changes were located in genes encoding proteins involved in synthesis of PG or associated structures. These include mutations in *lmo0421* encoding the RodA protein involved in cell wall elongation [25], *lmo1085* encoding the teichoic acid biosynthesis protein B (TagB) [26], *lmo1438* encoding the penicillin binding protein PBP B1, a transpeptidase involved in cell elongation [27], *murB*, which is crucial for synthesis of lipid II [28] and a frameshift mutation in *mreB*, required for rod-shaped growth of bacteria [29]. Furthermore, various alterations were found in genes with unknown functions but which encode for PG-bound proteins or proteins with a cell wall binding domain such as *lmo0160*, *lmo0320*, *lmo0627*, *lmo1799*, *lmo2085* or *lmo2179*. Although we do not know which of the genetic changes may lead to altered or impaired protein function, the high number of changes in genes related to cell wall synthesis suggests the dispensability of several cell wall synthesis associated genes. To elucidate to which extent cell wall synthesis is inhibited in this stable L-form, the different steps of the PG synthesis pathway were analyzed.

### CBDP40-GFP does not bind to the L-form surface, but PG precursors are present

**Fig 2:**
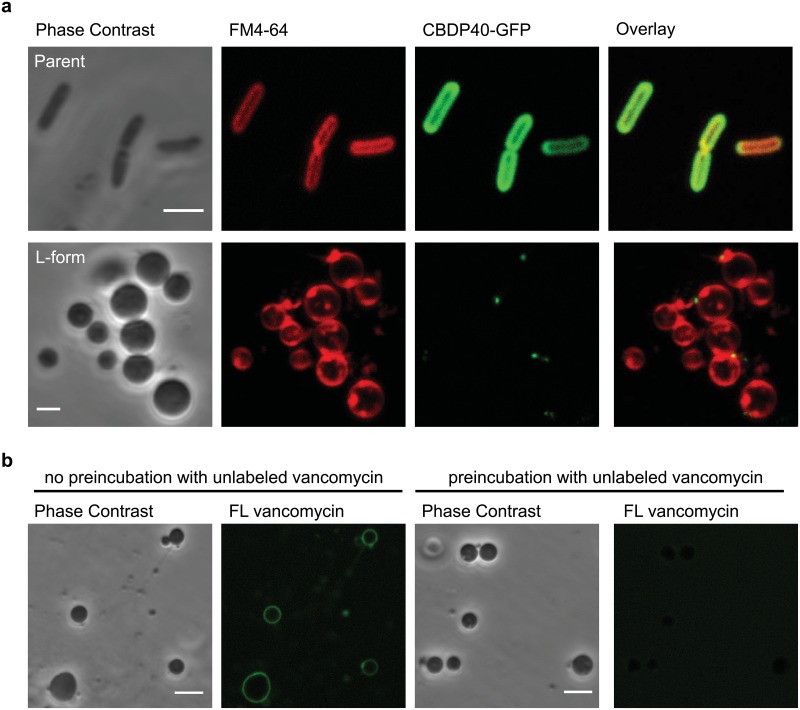
L-forms display PG precursors but not the binding sites of CBDP40-GFP on their surface. (a) Parent and L-form cells grown in soft agar were exposed to the PG affinity protein CBDP40-GFP. Whereas parent cell walls showed a clear decoration, no or only single spots of CBDP40-GFP were seen on the L-form surface. The membrane-specific dye FM 4–64 was used as a counterstain. Scale bars, 2 μm. (b) Fluorescently labeled vancomycin decorates the L-form cell surface. In contrast, no binding was detected when L-forms were preincubated with unlabeled vancomycin prior to FL vancomycin incubation. Scale bars, 4 μm.

To investigate the presence of cell wall remnants in the stable L-form strain, a GFP tagged bacteriophage derived cell wall binding protein CBDP40-GFP was used. CBDP40-GFP binds to PG itself, and not to PG associated molecules such as teichoic acids. It is not known to which specific moiety of the peptidoglycan (glycan, stem peptide or crosslink) CBDP40-GFP binds, but the fluorescent protein can be used to visualize the peptidoglycan sacculus or fragments thereof that are chemically identical to the peptidoglycan in rod-shaped cells, i.e. a fully polymerized peptidoglycan with directly cross-linked glycan chains (chemotype A1γ) [30, 31]. Incubation of CBDP40-GFP with the walled parental cells resulted in a strong fluorescence surrounding the cell, whereas no or only a few fluorescent spots could be observed on the L-form surface (Fig 2a). This fluorescence was located in areas with excess membrane material as indicated by a more intense staining with the membrane dye FM 4–64 (Fig 2a). In addition, fluorescent spots also occurred on L-form cells grown in presence of ramoplanin and penicillin G, which effectively inhibits cell wall assembly (data not shown). This suggests that the occasional fluorescent spots on the L-form surface are unspecific. These observations demonstrate that either no matured PG sacculus or fragments thereof are present, or if any PG is present, that it is chemically different from the wild type PG. Chemical modifications due to altered expression or mutations may shield access to PG for binding. However, given the spherical shape of the cells, which range in diameter from 0.5 μm to 30 μm, and the high sensitivity to changes in osmolarity (Fig 1a), an intact but chemically modified sacculus is unlikely.

To analyze if PG precursor synthesis is still present and functional, L-forms were incubated with fluorescein-conjugated vancomycin (FL vancomycin), which specifically binds to the D-alanyl-D-alanine terminus of the non-crosslinked stem peptide [32]. A strong and homogenous signal surrounding the cell was observed in L-forms (Fig 2b), indicating the presence of lipid II and/or nascent pentapeptide chains in PG. When L-forms were incubated with an excess of non-fluorescent vancomycin prior to FL vancomycin exposure in order to saturate the terminal D-ala-D-ala residues, no binding of FL vancomycin was detected (Fig 2b), indicating specificity of the fluorescent indicator.

### Bifunctional PG synthases are expressed in L-form cells

**Fig 3:**
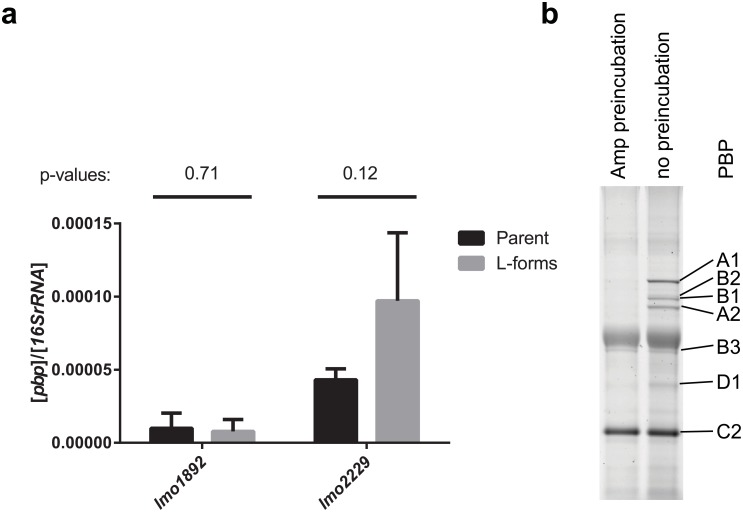
L-forms express both bifunctional PBPs. (a) Transcription levels of *lmo1892* (encoding PBP A1) and *lmo2229* (encoding PBP A2) are not significantly different in parental and L-form cells as determined by quantitative real-time PCR. Means and standard deviations of three biological replicates (n = 3), as well as pairwise t-tests and the corresponding P-values are depicted. (b) Incubation of total cell extracts of L-form *L*. *monocytogenes* with fluorescent BOCILLIN FL reveals the presence of the two bifunctional PBPs, PBP A1 and A2, and the transpeptidases PBP B1, B2 and B3. In addition, PBP C2, containing a β-lactamase class C domain, and PBP D1, containing a DD-carboxypeptidase domain, were detected. Samples preincubated with high concentrations of ampicillin serve as specificity control of BOCILLIN FL binding. PBP C2 and B3 were already reported to be poorly competed by ampicillin [33].

The FL vancomycin staining cannot differentiate between lipid II or uncrosslinked PG polymers. PG polymers are generated by PG synthases, also known as bifunctional PBPs, which contain a glycosyl transferase (GT) and a transpeptidase domain (TP). Thus the presence of these enzymes in the stable *Listeria* L-form was investigated. In *L*. *monocytogenes*, two bifunctional PBPs exist, PBP A1 (encoded by *lmo1892*) and PBP A2 (encoded by *lmo2229*) [33]. Genome sequencing revealed no mutations in the respective genes for these PBPs (S1 Table). Quantitative analysis of transcription of *lmo1892* and *lmo2229* by qPCR also demonstrated no significant difference in the transcription levels of both PBP genes in L-forms compared to parental cells (Fig 3a).

To determine if PBPs are actually produced in L-forms a BOCILLIN FL binding assay was performed using total cell extracts of *Listeria monocytogenes* L-form cells. BOCILLIN FL is a fluorescently labeled β-lactam antibiotic that specifically binds to transpeptidases, including the bifunctional PBPs, and thus can be used for their identification and quantification [34]. In the assay, bacterial cell extracts were first separated by ultracentrifugation, and the membrane extracts subsequently incubated with BOCILLIN FL to visualize the PBP profile by fluorescence imaging. Interestingly, seven of the ten known PBPs of *L*. *monocytogenes* could be detected, among them both bifunctional PBPs, PBP A1 and PBP A2 (Fig 3b).

### Glycosyl transfer in *Listeria* L-forms

**Fig 4:**
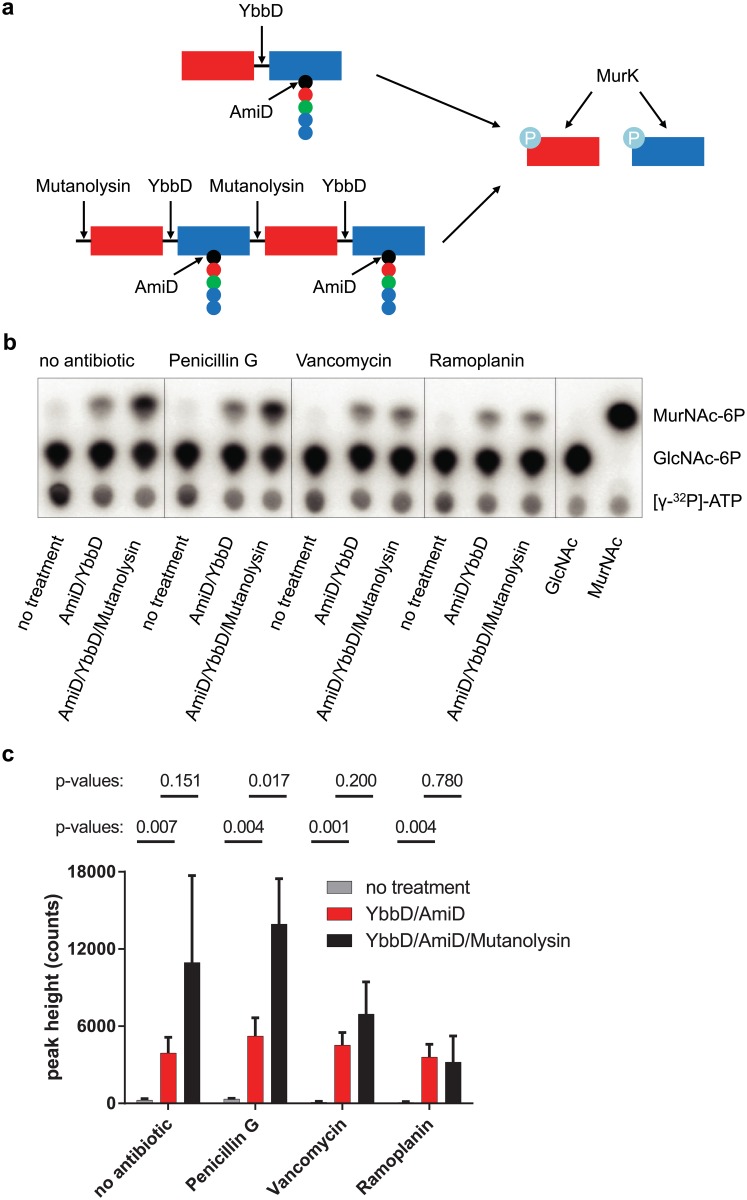
L-forms assemble PG strands. (a) Scheme showing the principle of the kinase assay. The amidase AmiD and the N-acetylglucosaminidase YbbD can be used to release GlcNAc (red rectangle) and MurNAc (blue rectangle) from GlcNAc-MurNAc disaccharides. Polymerized PG needs to be treated in addition with the muramidase mutanolysin in order to release the monosaccharides. Released GlcNAc and MurNAc can subsequently be phosphorylated by the kinase MurK using radioactively labeled substrate, followed by detection using thin layer chromatography. The dashed arrows indicate the cleavage sites of the respective enzymes. The five circles represent the stem pentapeptide of MurNAc, which is cleaved by AmiD. (b) Thin layer chromatography of radio-labelled enzymatic digestion products. The labels above the chromatogram depict the specific antibiotics in whose presence the L-forms were grown. The labels below the chromatogram depict with which enzymes the extracts were treated before the kinase assay. Cell extracts of L-forms treated with the kinase MurK in presence of [γ^32^P]-ATP display a radioactive signal corresponding to MurNAc-6P after separation by chromatography. The MurNAc-6P increases continuously when the samples are pretreated with AmiD and YbbD or mutanolysin in combination with AmiD and YbbD. This indicates the presence of longer PG strands in L-forms. Extracts from L-forms grown in presence of various antibiotics were used as control for the specificity of the assay. (c) Quantification of the MurNAc-6P signal of three independent measurements by density plotting. Means and standard deviations of three biological replicates (n = 3), as well as pairwise t-tests and the corresponding P-values are depicted.

Based on the presence of at least lipid II and both *Listeria* bifunctional PBPs in the L-forms we speculated that PG polymers are synthesized, especially since *in vitro* experiments showed successful PG assembly with just lipid II and bifunctional PBPs [35, 36]. To test for the presence of PG strands in L-form cells, a novel sensitive assay to detect glycosyl transferase activity was employed [37]. This assay makes use of the *Clostridium acetobutylicum* kinase MurK. This enzyme specifically phosphorylates N-acetyl muramic acid (MurNAc) and N-acetyl glucosamine (GlcNAc) using ATP as phosphate source [38]. MurK in combination with radioactively labeled [γ^32^P]-ATP can be used to label free GlcNAc and MurNAc, which can subsequently be detected using thin-layer chromatography. Using different sets of enzymes that release MurNAc either from disaccharide-pentapeptides or from PG strands, and using different antibiotics, the presence of different forms of MurNAc can be detected. The principle of the assay, including the target sites of all enzymes used is depicted in Fig 4a. Fig 4b shows one replicate of the thin-layer chromatography, and Fig 4c the quantification of three independent assays. Analysis of L-form extracts treated with MurK in presence of [γ^32^P]-ATP revealed only very low amounts of free MurNAc. GlcNAc was present, but also in the medium control (S1a Fig), making any conclusion impossible regarding GlcNAc. When L-form extracts were pretreated with a β-N-acetylglucosaminidase (YbbD) [39] and a muramyl amidase (AmiD) [40], an additional strong signal corresponding to MurNAc-6-phosphate appeared (Fig 4b). This implies the presence of at least the disaccharide units GlcNAc-MurNAc-(peptide) (Fig 4a), as simultaneous digestion with YbbD and AmiD releases more MurNAc residues than in the undigested sample. This signal may originate from turnover products of the PG recycling pathway present in the cytoplasm since a MurK treated lipid II control does not yield a MurNAc signal (S1c Fig). When the L-form extracts were additionally treated with mutanolysin, which cuts the β-N-acetylmuramyl-(1→4)-N-acetylglucosamine linkage, the MurNAc-6P signal increased further (Fig 4b and 4c). This finding indicates the presence of polymerized PG strands in the L-forms, from which more MurNAc residues can be released after mutanolysin digestion (Fig 4a). Similar observations were obtained with walled *L*. *monocytogenes* cells that also showed the same increase in the MurNAc-6P signal, when treated with the different enzymes (S1b Fig), demonstrating the capacity of the assay to detect polymerized PG. As a control, L-forms grown in presence of penicillin G, vancomycin or ramoplanin were analyzed with the same assay. L-forms grown in presence of penicillin G showed a similar pattern for the MurNAc-6P signal as the sample grown in absence of antibiotics, which was expected since penicillin G specifically inhibits transpeptidation only and therefore PG polymerization should still be observed. Cells grown in presence of ramoplanin, however, showed no increase of the MurNAc-6P signal after mutanolysin treatment, since ramoplanin is a specific inhibitor of the glycosyl transferase reaction and therefore no PG polymerization should occur. Vancomycin, which inhibits both glycosyl transfer and transpeptidation, had an intermediate effect as only a slight increase of the MurNAc-6P signal after mutanolysin treatment was observed. In conclusion, our data show that the assay is in fact specific for the different steps in PG assembly and indicates that PG strands are polymerized in the stable *L*. *monocytogenes* L-form.

### L-forms do not revert to the walled state despite complementation of genes involved in late steps of cell wall assembly

The last step during cell wall synthesis is crosslinking of the nascent PG strands. The sequencing results reveal a point mutation in *lmo1438*, encoding the transpeptidase PBP B1, which is directly involved in crosslinking. In addition, two genes important for rod-shaped growth of bacteria are also mutated, *lmo0421* (encoding RodA) and *mreB*. Since we already showed the synthesis of PG strands in the stable L-form, we reasoned that the mutation causing the stable L-form phenotype has to be involved in the final step of cell wall assembly, specifically crosslinking. The mutations in *lmo0421*, *lmo1438* or *mreB* were considered as potential candidates. Therefore, a complementation study was performed. We generated derivatives of the stable L-form strain expressing either a wt copy of one of the three genes, a combination of two wt copies out of the three genes, or all three wt copies of the genes in *trans* (S2 Table). The complemented strains were then plated onto DM3 medium, which was reported to support the reversion of bacterial protoplasts [41] and also supports reversion of transient, unstable *L*. *monocytogenes* L-forms (data not shown). However, none of the complemented strains was able to revert to the rod-shaped form, suggesting that the stable phenotype results here from the multitude of mutations rather than from one specific mutation.

## Discussion

The traditional classification of L-forms into stable and unstable types is based on their potential to revert to intact, walled bacteria. It has been recently demonstrated that unstable *E*. *coli* L-forms still show residual septal PG synthesis, which is essential for L-form multiplication and which acts as a starting point for new PG synthesis covering the complete bacterium from the onset of reversion [2–4]. Stable L-forms, on the contrary, are proposed to lack a complete cell wall due to genetic changes that impair PG synthesis [14]. Kawai et al. (2014) have shown that stable L-forms can resynthesize a cell wall without the need for a pre-existing template, when absent PG precursor synthesis genes (*murC*, *uppS*) are provided again in *trans*, further corroborating the hypothesis that genetic changes and not the lack of template PG are blocking reversion in stable L-forms [13]. L-forms lacking essential components of the PG precursor synthesis pathway can be constructed with molecular techniques and are viable, showing that PG synthesis is completely dispensable in at least these stable L-forms [6].

We demonstrate here that stable L-forms generated by prolonged exposure to penicillin G can still continue PG synthesis without reaching a fully matured cell wall sacculus. Since the here studied L-form still polymerizes PG, correct crosslinking of the PG strands may not take place any longer, explaining the lack of sacculus formation. This might also explain the absence of CBDP40-GFP binding, which might specifically target the crosslink of peptidoglycan or may require the specific three dimensional PG configuration that results from crosslinking. Noteworthy, the observed residual PG synthesis seems dispensable as both glycosyl transfer and transpeptidation can be inhibited by high concentrations of antibiotics (25x MIC) without affecting L-form viability, further supported by the ability of the D-Ala auxotroph to grow and multiply in the L-form state. Nevertheless, it cannot be fully excluded that the antibiotics still allow some minor PG synthesis, which is sufficient and essential for growth and survival. With regard to the ongoing precursor synthesis, it is possible that it remains essential in this model of stable L-forms. However, we suggest that it is rather a defective, remaining process of the previously intact PG synthesis pathway and does not serve any functional role such as providing osmotic stability or determination of the cell shape. Full maturation appears to be hindered by at least one but likely many mutations which have been accumulated under penicillin G selection pressure in osmoprotective medium, without affecting PG precursor synthesis and glycosyl transfer. Given the randomness of mutations and the dispensability of glycosyl transfer in our model, it is possible that when a new strain would be generated with the same procedure, also the glycosyl transfer step could be affected.

Recent studies revealed that several proteins essential in the walled state become dispensable in L-forms [6, 7, 10]. A prominent example is FtsZ, which is involved in recruiting the division machinery to the future division site and is found in most bacteria [42]. Therefore, prolonged L-form passaging in absence of a cell wall permits the accumulation of genetic changes in otherwise essential genes in a time-dependent manner. This may have also occurred in the *Listeria* L-form strain studied here, where various mutations have been identified (S1 Table), many of them in genes which clearly play an important role during cell wall assembly. However, it is not clear, which of these genetic changes actually result in proteins with impaired or altered functions. For example, the point mutation in the essential *murB* gene (S1 Table) does not impair the function of the MurB protein, as PG precursor generation could still be observed (Fig 2b). Also the mutation in *lmo1438*, encoding PBP B1, does at least not inhibit its substrate binding capacities, as it could still be detected in the BOCILLIN FL binding assay (Fig 1b). However, active PBPs do not necessarily imply that they are functional as they require other factors such as spatial location and protein-protein interactions (e.g. type B HMM-PBPs act in close cooperation with type A HMM-PBPs). *MreB* has a frameshift early in the open reading frame (position 117/1014 bp), most likely resulting in a complete loss-of-function. Complementation of the mutant *lmo1438*, *rodA* or *mreB* genes, all involved in the later stages of cell wall assembly, did not enable reversion of the stable L-form to the walled state. One explanation could be a dominant negative effect of the mutated gene products, which can only be overcome by gene replacement with the wt gene and not with overexpression of the wt gene in *trans*. However, gene replacement is not possible in this stable L-form due to a pronounced polyploidy (P. Studer, unpublished data). Alternatively, one or more mutations in one of the other genes prevent reversion to the walled form. Importantly, it was shown that proteins related to PG synthesis which are not essential for proliferation of walled cells may become essential for *de novo* cell wall synthesis in cells that have no or a damaged cell wall. Examples are the Rcs cell wall stress response proteins, the penicillin binding protein PBP1B and the lipoprotein LpoB, all of which, although otherwise nonessential, become essential for reversion of *E*. *coli* protoplasts that have been generated by lysozyme treatment [43]. Furthermore, certain metabolic changes that are required for proliferation in the L-form state, such as enhanced membrane synthesis and fluidity, may be incompatible with growth in the walled state, thereby preventing reversion to the walled form [9].

The fate of the produced PG strands is unclear and remains to be elucidated. It is known that during bacterial proliferation up to 50% of the PG of a bacterium is turned over. This PG is either released into the environment, recycled by the cell and used for synthesis of new PG, or degraded and used for other metabolic processes [44, 45]. It is conceivable that the PG intermediates synthesized by L-forms may also be either degraded or recycled and reused, the latter of which would energetically be much more efficient.

A controversial, yet highly relevant topic is the putative role of L-forms in disease, especially during persistent bacterial infections [46–48]. Several studies report apparent cell wall-deficient bacteria isolated from diseased humans [48]. In some cases the bacteria were identified as *L*. *monocytogenes* [49, 50]. However, these data are based on single, unrelated case studies. We have recently shown that stable *L*. *monocytogenes* L-forms are essentially non-pathogenic and are efficiently cleared by both preactivated and bone marrow derived macrophages [51]. In addition, the L-form cells used in this study are stable and thus not able to revert to the pathogenic walled form. Thus the conversion from transient to stable L-forms may represent the point-of-no-return with respect to bacterial virulence; however, further research will be needed. The finding that even stable L-forms are still able to produce PG strands, as we have observed in the present study, suggests that L-forms may still be recognized by the host in a similar manner as their walled counterparts. From this new perspective, the suggested selective advantage over the walled bacterium with respect to immune activation and recognition should be nuanced.

In conclusion, we have shown that the absence of a mature cell wall sacculus in a stable L-form strain does not necessarily imply the absence of PG synthesis, which is in contrast to the intuitive, commonly accepted view. Specifically, PG precursors are still produced and glycosyl transferase activity resulting in the synthesis of glycan strands has been demonstrated. Continued cultivation of the here studied L-form strain is expected to lead to even more genetic alterations in pathways dispensable for the L-form state. Since we have shown that inhibition of the residual PG synthesis by antibiotics does not affect L-form viability and that *Listeria* L-forms deficient in PG precursor synthesis are viable, these genetic changes may at one point result in the loss of the complete PG pathway.

## Methods

### Bacterial strains and growth conditions

*Listeria monocytogenes* EGDe was used for all experiments except for the D-alanine auxotrophy experiments, which were performed with *L*. *monocytogenes* strain DP-L3506 [23]. Both strains were grown at 30°C in BHI medium unless stated otherwise, supplemented with 0–100 μg ml^-1^ D-alanine for DP-L3506.

### L-form induction, stabilization and growth

The novel L-form strain used in this study was generated essentially as described previously [8]. Briefly, an overnight culture of walled *L*. *monocytogenes* EGDe was inoculated into *Listeria* L-form medium (LLM) soft agar (37 g l^-1^ BHI, 150 g l^-1^ sucrose, 2.5 g l^-1^ MgSO_4_ x 7 H_2_O, 3 g l^-1^ milk serum powder, 3 g l^-1^ agar) supplemented with 50 μg ml^-1^ penicillin G. After several days to weeks, developing colonies were isolated and further cultivated in LLM soft agar tubes with penicillin G. The resulting transient L-forms were then stabilized by gradually reducing the penicillin G concentration in the soft agar (50, 25, and 12.5 μg ml^-1^) during passaging. After approximately two years of passaging in LLM soft agar in the absence of penicillin G, an LLM liquid culture was inoculated with colonies from the soft agar and growth could be observed after 1–2 weeks. After two more passages in order to adapt the L-form cells to the liquid medium, they were surface-plated onto LLM agar plates (LLM with 1% agar). A single colony was then inoculated into liquid medium, and a glycerol stock was prepared from this culture. From the time point where L-forms were grown in liquid culture, the 3 g l^-1^ of milk serum powder in the *Listeria* L-form medium were exchanged by 1% of heat-inactivated horse serum (Sigma) and all experiments were performed with the new LLM formulation (37 g l^-1^ BHI, 150 g l^-1^ sucrose, 2.5 g l^-1^ MgSO_4_ x 7 H_2_O, 1% heat-inactivated horse serum). Induction of the D-Ala auxotrophic strain DP-L3506 [23] was done in the same *Listeria* L-form medium. An overnight culture in BHI medium (Biolife, Italy) in presence of 100 μg/ml D-Ala was extensively washed with LLM and an amount of 100 μl was inoculated in LLM soft agar without penicillin G. Protoplasts were spontaneously formed within 24 h and propagated after weekly subcultivation in LLM soft agar without penicillin G or D-Ala. L-forms were always cultivated statically in LLM at 32°C.

### Sensitivity to changes in osmolarity

L-forms grown for 2 d in liquid LLM and walled *Listeria* grown overnight in LLM were used to determine their sensitivity to low osmotic conditions. Briefly, 10 μl of a 2 d old L-form culture or a 1:10 diluted overnight culture of walled *Listeria* were added to 990 μl deionized water or LLM as control. The samples were mixed by vortexing and incubated for 5 min at room temperature. Subsequently, a 10-fold dilution series in LLM was prepared and 20 μl of every dilution was spotted onto LLM plates. The plates were sealed with parafilm and incubated at 32°C for 1 d for walled *Listeria* or for 9 d for L-forms, before being imaged.

### Genome sequencing

Twelve 4 ml LLM liquid cultures with 3 d old L-forms were pooled in a falcon tube, centrifuged at 12’000 g for 5 min and resuspended in 40 ml L-form washing buffer (150 g l^-1^ sucrose, 2.5 g l^-1^ MgSO_4_ x 7 H_2_O, 7.59 g l^-1^ NaCl). After another centrifugation step at 12’000 g for 5 min, the pellet was resuspended in 200 μl PBS and the DNA was extracted with the GenElute Bacterial Genomic DNA Kit (Sigma Aldrich) according to the manufacturer’s instructions. Library preparation and sequencing was performed by the Functional Genomics Center Zurich on a Pacific Biosciences RS system.

### CBDP40-GFP staining

A volume of 38 μl L-form cells cultivated for 1 d, and the same volume of late log-phase growing parent *Listeria* cultivated in LLM soft agar at 32°C were mixed with 2 μl CBDP40-GFP (1.6 mg ml^-1^). After 15 min incubation at room temperature, 1 ml liquid LLM was added and the cells were washed twice with 1 ml liquid LLM (10’000 g, 2 min). The final pellet was resuspended in 20 μl liquid LLM. Thereof, 8 μl was mixed with 2 μl FM 4–64 (200 μg ml^-1^) and subsequently imaged using confocal fluorescence microscopy.

### Vancomycin FL labeling

One day old L-forms grown in soft agar were incubated with either unlabeled vancomycin (1 mg ml^-1^ final concentration) (specificity control) or the same amount of deionized water for 15 min at room temperature. Then, 9 μl of each sample was mixed with 1 μl of vancomycin FL (200 μg ml^-1^; Life Technologies) and directly imaged by confocal fluorescence microscopy.

### RNA extraction, Reverse Transcription and Quantitative real-time PCR

For *lmo1892* (encodes PBP A1) and *lmo2229* (encodes PBP A2) expression analysis, the total RNA was extracted from 3 d old L-forms grown in LLM soft agar and exponential phase parent cells (OD_600nm_ = 0.4–0.45) grown in LLM medium at 32°C. For parent cells, 25 ml cultures were used for RNA extraction and for L-forms the content of three soft agar tubes was combined. Cells were mechanically broken by bead-beating and RNA was purified using a standard phenol-chloroform extraction, followed by precipitation with isopropanol. DNA was removed by DNase (5 PRIME) treatment for 30 min at room temperature. The RNA was further purified with the RNeasy Mini Kit (Qiagen) according to the manufacturer’s instructions with the exception, that an additional on-column DNase treatment (5 PRIME) was performed. A PCR was used to check for residual, contaminating DNA. In case DNA was found to be present, the purification was repeated with the RNeasy Mini Kit.

Synthesis of cDNA from pure RNA samples was performed using the TaqMan^®^ Reverse Transcription Reagents (Roche Molecular Systems). Therefore, 350 ng RNA was mixed with 5 μl 10X TaqMan RT Buffer, 11 μl 25 mM magnesium chloride, 10 μl dNTPs mixture (2.5 mM each), 2.5 μl random hexamers, 1 μl RNase inhibitor and 1.25 μl MultiScribe reverse transcriptase (50 U μl^-1^). Reverse transcription was performed in a thermo cycler using the following conditions: 25°C for 10 min, 48°C for 30 min and 95°C for 5 min.

The SYBR Green PCR Master Mix from Applied Biosystems was used for qPCR measurement on a Rotor-Gene 6000 (Corbett Life Science) device with the following cycling conditions: 95°C for 10 min, 50 x (95°C for 10 s, 60°C for 15 s, 72°C for 20 s), followed by a melting curve analysis. The reaction mixture was composed of 5 μl SYBR Green, 1 μl of forward and reversion primer (each 4 μM), 1 μl deionized water and 2 μl of the measured sample. The primers used for measurement of *lmo1892* copies were PBP A1-qF (5’–TGC CCA AGA ATA TAC GGA GA– 3’) and PBP A1-qR (5’–ATT GCT TGA ACA CGT CCA GT– 3’), for *lmo2229* the primers were PBP A2-qF (5’–AAA GGT GCT TCG CCA GTA GA– 3’) and PBP A2-qR (5’–GCC TGA TGG ATC GAC AAT TT– 3’). The *16SrRNA* housekeeping gene was used for internal normalization and was amplified with the primers 16SrRNA_fwd_RT3 (5’–GAT GCA TAG CCG ACC TGA– 3’) and 16SrRNA_rev_RT4 (5’–GCT CCG TCA GAC TTT CGT C– 3’). Because the copy numbers of the *16SrRNA* gene were extremely high, the samples were 1:1000 diluted prior to measurement of the *16SrRNA* copy number. The experiments were performed with three biological replicates and every sample was in addition measured in duplicates. Furthermore a standard curve was prepared in every qPCR run, consisting of a ten-fold dilution series starting from 10^8^ down to 10^1^ copies μl^-1^ of *L*. *monocytogenes* EGDe chromosomes.

### BOCILLIN FL assay

L-form cells were grown for 2 d in soft agar at 32°C, and parent cells for 15 h at 32°C in soft agar. Then, the content of nine soft agar tubes was pooled (approximately 4 ml in total) and cooled on ice for 10 minutes. Subsequently 4 ml of 50 mM sodium phosphate buffer (pH 7.5) was added and the samples were vortexed and sonicated for 5 min with 50% amplitude and 40% power with a SONOPULS HD 2070 sonicator (Bandelin). The extracts were then frozen at -80°C until being used for further analysis. For the BOCILLIN FL assay the cells were thawed and sonicated on ice at maximal intensity for 5 min with a small tip on a LABSONIC^R^ equipment (SARTORIUS). After centrifugation at 70,000 rpm for 30 min in a Beckman TL-100 ultracentrifuge, the supernatant was removed and the pellets resuspended in 400 μl 50 mM sodium phosphate buffer (pH 7.2), and 100 μl were labeled by incubation at 37°C for 30 min with a concentration of 20 μM BOCILLIN FL (Molecular Probes). After binding, loading buffer NuPAGE 4x was added and samples boiled for 10 min. After centrifugation at 14,500 rpm for 5 min in a minispin eppendorf, 50 μl of each sample was loaded on a NuPAGE 8% Bis-Tris gel (Invitrogen) and run in MOPS buffer at 80 V. Competition experiments were carried out by preincubation of the samples with ampicillin (200 μg/ml) for 30 min at 37°C, before adding the fluorescent antibiotic. The samples were then further incubated for 30 min at 37°C in the presence of BOCILLIN FL 20 μM. PBPs were visualized directly on the polyacrylamide gel by fluorescence using a Typhoon 9410 imager (General Electric) with excitation wavelength of 488 nm and emission filter 520BP40 for BOCILLIN FL.

### Kinase assay

L-forms were grown at 32°C for 7 d in LLM soft agar supplemented with either 50 μg ml^-1^ penicillin G sodium salt (Sigma-Aldrich), 100 μg ml^-1^ vancomycin hydrochloride (Calbiochem), 6.25 μg ml^-1^ ramoplanin (Sigma-Aldrich) or without antibiotics. Colonies in the same growth phase were then transferred to an Eppendorf tube (200–600 μl per tube) and mixed with an equal volume of 10 mM Tris-HCl buffer with pH 7.0. The L-forms were heat-killed by incubation at 72°C for 10 min and stored at 4°C until being used for further analysis. Walled cells were isolated similarly but after one overnight growth at 32°C in LLM soft agar. Of each sample, 20 μl was incubated with 5 μg mutanolysin from *Streptomyces globisporus* (Sigma-Aldrich, 4000 U/mg) or with 5 μl water at 37°C for 24 h on a rotary shaker. After an enzyme inactivation step (10 min at 95°C), degraded PG of the L-forms was further incubated for 2 h at 37°C with two additional PG hydrolases (5 μg each). The PG hydrolases used were the N-acetyl muramic acid-L-Ala amidase AmiD from *E*. *coli*, as previously described [40], and the N-acetyl glucosaminidase YbbD (NagZ) from *B*. *subtilis*, prepared as described previously [39]. As a control, 5 μl of a 20 mM Na_2_HPO_4_, 500 mM NaCl buffer (pH 7.5) was used. After incubation, enzymes were inactivated (10 min at 95°C), samples were evaporated in a rotational vacuum concentrator (RVC 2–18, Christ, Osterode, Germany) at 40°C and stored until further analysis at 4°C. Dried pellets were then resuspended in 5 μl of 25 mM Tris buffer, pH 7.5 and 15 μl master mix (end concentration in 20 μl total volume of 25 mM Tris buffer, 10 mM MgCl_2_, 20 ng MurK from *Clostridium acetobutylicum* (as previously described [37, 38]) and 300 Bq [γ^32^P]-ATP) was added. As controls, 5 μl of GlcNAc from Sigma-Aldrich and MurNAc from Bachem, Bubendorf, Switzerland (5 mM each standard) were used, and incubated with the master mix as described for L-form digested samples. Reactions mixtures were incubated for 1 h at 37°C under constant shaking and 2 μl from each sample were spotted onto a thin-layer chromatography (TLC) plate (silica gel 60 F254 from Merck, Darmstadt, Germany). The components in the reaction mixture were separated by TLC using as a mobile phase butanol:methanol:ammonia:water (5:4:2:1) [vol/vol/vol/vol] solution. Radioactive samples were detected by autoradiography using a Typhoon Trio phosphoimager (GE Healthcare Life Science) and spot intensities were quantified by ImageQuant TL 7.0 Software.

For the lipid II control, 10 μl lipid II (100 μM, dissolved in water, DAP form, kindly provided by E.J. Breukink and obtained using enzymatic synthesis) was mixed with 10 μl MES buffer. Five microliter ddH_2_O or 5 μl mutanolysin (1 mg ml^-1^) were added and samples were incubated in a rotary shaker at 37°C for 20 h. The enzyme was inactivated for 10 min at 95°C. Five microgram AmiD and 5 μg YbbD or water were added and samples were incubated for 2 h at 37°C in a rotary shaker. Enzymes were inactivated (10 min at 95°C) and samples were dried for about 1 h at 40°C. Pellets were resuspended in 5 μl 25 mM Tris buffer, pH 7 (so lipid II has a final concentration of 200 μM) and stored overnight at 4°C. As standards 5 μl GlcNAc or MurNAc (each 200 μM) were used. Then, 15 μl master mix was added and samples were further treated as described above. After incubation with the MurK enzyme for 1 h at 37°C, 4 μl (2 x 2 μl) of the samples were loaded on the TLC plate.

### Microscopy

Confocal laser scanning microscopy was performed on a Leica TCS SPE system (Leica Microsystems GmbH, Wetzlar, Germany), equipped with a HCX PL FLUOTAR 100.0 x 1.30 OIL objective. FL vancomycin and CBDP40-GFP were excited with a 488 nm laser and the photomultiplier collected the light from 490–637 nm or 490–536 nm, respectively. The lipophilic membrane dye FM^®^ 4–64 was excited with the 532 nm laser and the photomultiplier collected the light from 600–799 nm. FM^®^ 4–64 (Life Technologies) was used at a final concentration of 40 μg ml^-1^. The cells were mixed with the dye and immediately observed without washing. The microscope pictures were prepared for publication using the software ImageJ (Version 1.48e, National Institutes of Health, USA).

### Construction of complementation strains

*lmo1438* was amplified from chromosomal *L*. *monocytogenes* EGDe DNA using primers lmo1438_XmaI_F (5’–TCC CCC CGG GCA AAC TAA ATT TTA GAA AAA AGA AAA AAG– 3’) and lmo1438_SalI_R (5’–TTT GTC GAC TTA ATT TTC GGT TTG TTC TGA TTG TGC– 3’). The product was digested with XmaI and SalI and ligated into pIMK3 which was digested with same enzymes. Wild-type *lmo0421* (RodA) was either amplified with the primers lmo0421_NcoI_F (5’–TTT CCA TGG AAA TGA GTT CTT CTA CAT TTG AAG– 3’) and lmo0421_SalI_R (5’–TTT GTC GAC TTA ATT AAC CAG TGT AGA CTC TAC– 3’), digested with NcoI and SalI and ligated into digested pIMK3 vector, or amplified with the primers PS20 (5’–GAA GGA GAG TGA AAC CCA TGA GTT CTT CTA CAT TTG AAG– 3’) and PS23 (5’–CGA CTC GAG TCT AGA TTA ATT AAC CAG TGT AGA CTC TAC– 3’) and inserted by Gibson assembly into the plasmid pLEB579[52] harboring the constitutive P_help_ promoter [53]. *mreB* was amplified with primers mreB_KpnI_F (5’–TTT GGT ACC AGG AGA ATA CAG ATG TTT GGA TTT GG– 3’) and mreB_PstI_R (5’–TTT CTG CAG TTA GTT CAT TTT TTT ACG TTT ATA CAT ATC– 3’), digested with KpnI and PstI and ligated into the digested plasmid pLEB579 harboring the inducible rhamnose promoter. To construct a vector that expresses both *mreB* and *lmo0421*, we amplified P_help_-lmo0421 from pLEB579/P_help_-lmo0421 using primers PS55 (5’–TAA AAA AAT GAA CTA ACT GCA GGA TCC CAT TAT GCT TTG– 3’) and PS57 (5’–GCG CGC GAT ATC TCT AGA TTA ATT AAC CAG TGT AGA CTC– 3’) and assembled it using Gibson assembly with the vector pLEB579/P_rha_-mreB, which was linearized by PCR using primers PS56 (5’–AAG CAT AAT GGG ATC CTG CAG TTA GTT CAT TTT TTT ACG– 3’) and PS58 (5’–TCT ACA CTG GTT AAT TAA TCT AGA GAT ATC GCG CGC TAT– 3’). L-forms were transformed with the constructs by electroporation. All steps for preparation of competent L-forms were performed on ice or in a microcentrifuge that was cooled to 4°C. Briefly, 4 ml of a three day old L-form liquid culture was washed two times with 2 ml sucrose-glycerol wash buffer (SGBW; 10% glycerol, 500 mM sucrose; pH adjusted to 7 with 100 mM NaOH; filter sterilized; [54]). All centrifugation steps were performed for 5 min with 5’000 g. The final pellet was resuspended in 50 μl SGWB, mixed with 10 μl of plasmid and used for electroporation with 10 kV cm^-1^ (400 Ω, and 25 μF). L-forms were recovered in 1 ml LLM for 4 h at 32°C before being inoculated into LLM supplemented with 12.5 μg ml^-1^ kanamycin for selection of the pIMK3 constructs or 5 μg ml^-1^ erythromycin for selection of the pLEB579 construct.

## Supporting Information

S1 Fig Control experiments of the kinase assay. (a) *Listeria* L-form medium alone without any bacteria yields a strong GlcNAc-6P signal, when exposed to the kinase MurK in presence of radiolabeled ATP for 30 min. (b) Walled *L*. *monocytogenes* were analyzed for the presence of polymerized peptidoglycan strands. Untreated extracts of walled *L*. *monocytogenes* exposed to MurK in presence of radiolabeled ATP did not result in a MurNAc-6P signal following thin-layer chromatography and detection by autoradiography. When the cell extracts were treated with the amidase AmiD and the glucosaminidase YbbD before exposure to MurK and radiolabeled ATP, a MurNAc-6P signal emerged, which indicates the presence of the disaccharide units GlcNAc-MurNAc-(peptide). This MurNAc-6P signal increased further when the cell extracts were additionally treated with mutanolysin before radio-labeling, confirming the presence of long peptidoglycan strands in walled *L*. *monocytogenes* cells. (c) Lipid II does not result in a MurNAc-6P signal in the kinase assay, neither when untreated, nor when pretreated with AmiD and YbbD, or mutanolysin in combination with AmiD and YbbD. GlcNAc and MurNAc alone are shown as standards.
(DOC)

S1 Table Genetic changes in the stable *L*. *monocytogenes* L-form. The base positions refer to the position in the annoted NCBI *L*. *monocytogenes* EGDe reference sequence (NC_003210). The gene products are derived from the NCBI Gene database. SNP = single nucleotide polymorphism, Ins = insertion, Del = deletion, fs = frameshift.
(DOC)

S2 Table The stable *L*. *monocytogenes* L-form was transformed with the following combinations of plasmids in order to test for reversion to the rod shape. Prha = rhamnose inducible promoter, Phelp = highly expressed, constitutive promoter.
(DOC)
