# Supplementary material for: The Absence of a Mature Cell Wall Sacculus in Stable Listeria monocytogenes L-Form Cells Is Independent of Peptidoglycan Synthesis
Source: PLoS One. 2016 May 5;11(5):e0154925. doi: 10.1371/journal.pone.0154925 (PMC4858229; doi:10.1371/journal.pone.0154925)
Supplement: S1 Fig — (a) Listeria L-form medium alone without any bacteria yields a strong GlcNAc-6P signal, when exposed to the kinase MurK in presence of radiolabeled ATP for 30 min. (b) Walled L. monocytogenes were analyzed for the presence of polymerized peptidoglycan strands. Untreated extracts of walled L. monocytogenes exposed to MurK in presence of radiolabeled ATP did not result in a MurNAc-6P signal following thin-layer chromatography and detection by autoradiography. When the cell extracts were treated with the amidase AmiD and the glucosaminidase YbbD before exposure to MurK and radiolabeled ATP, a MurNAc-6P signal emerged, which indicates the presence of the disaccharide units GlcNAc-MurNAc-(peptide). This MurNAc-6P signal increased further when the cell extracts were additionally treated with mutanolysin before radio-labeling, confirming the presence of long peptidoglycan strands in walled L. monocytogenes cells. (c) Lipid II does not result in a MurNAc-6P signal in the kinase assay, neither when untreated, nor when pretreated with AmiD and YbbD, or mutanolysin in combination with AmiD and YbbD. GlcNAc and MurNAc alone are shown as standards. (DOC) [file pone.0154925.s001.doc]

**
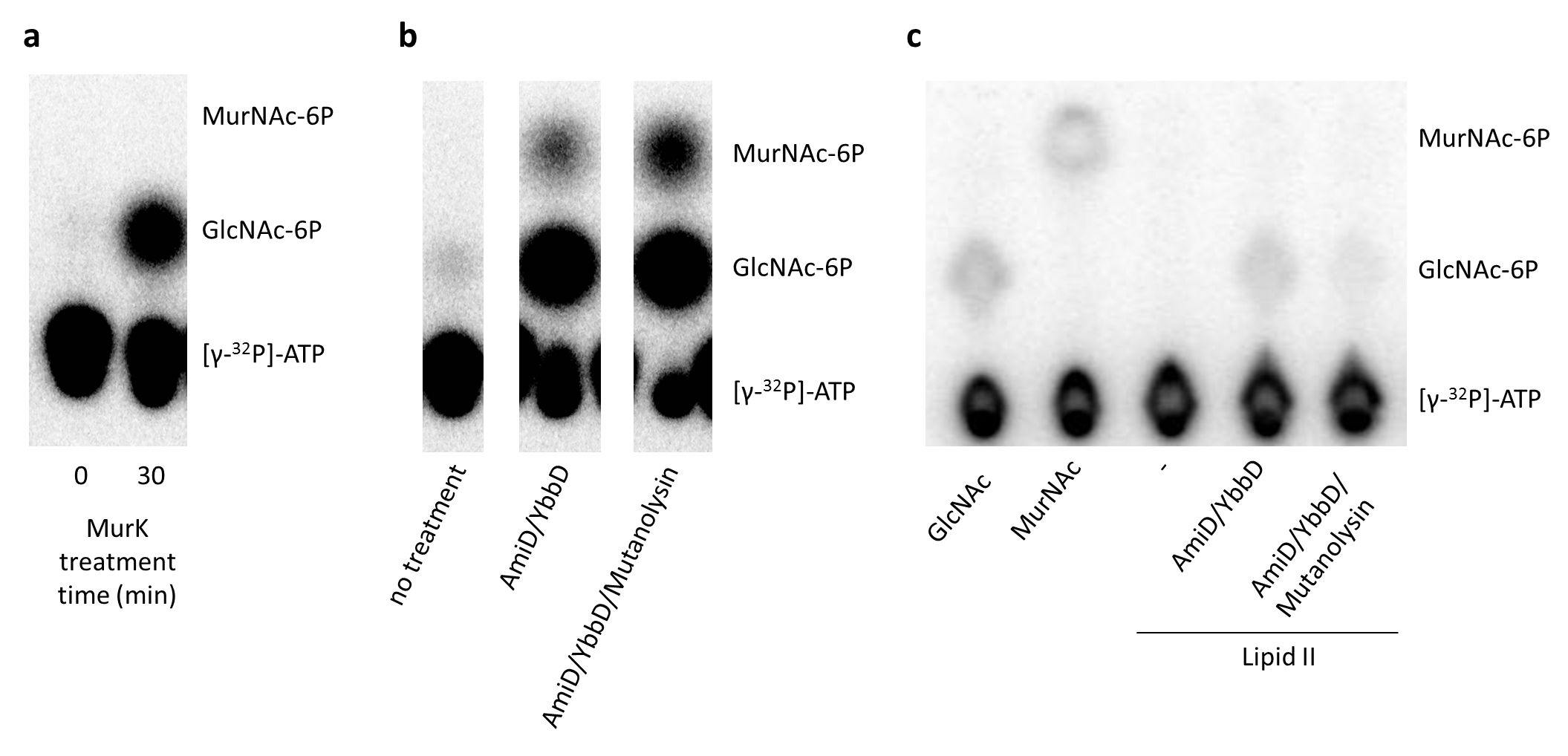
**

**S1 Fig. Control experiments of the kinase assay.** (a) *Listeria* L-form medium alone without any bacteria yields a strong GlcNAc-6P signal, when exposed to the kinase MurK in presence of radiolabeled ATP for 30 min. (b) Walled *L. monocytogenes* were analyzed for the presence of polymerized peptidoglycan strands. Untreated extracts of walled *L. monocytogenes* exposed to MurK in presence of radiolabeled ATP did not result in a MurNAc-6P signal following thin-layer chromatography and detection by autoradiography. When the cell extracts were treated with the amidase AmiD and the glucosaminidase YbbD before exposure to MurK and radiolabeled ATP, a MurNAc-6P signal emerged, which indicates the presence of the disaccharide units GlcNAc-MurNAc-(peptide). This MurNAc-6P signal increased further when the cell extracts were additionally treated with mutanolysin before radio-labeling, confirming the presence of long peptidoglycan strands in walled *L. monocytogenes* cells. (c) Lipid II does not result in a MurNAc-6P signal in the kinase assay, neither when untreated, nor when pretreated with AmiD and YbbD, or mutanolysin in combination with AmiD and YbbD. GlcNAc and MurNAc alone are shown as standards.
