# Supplementary material for: The Absence of a Mature Cell Wall Sacculus in Stable Listeria monocytogenes L-Form Cells Is Independent of Peptidoglycan Synthesis
Source: PLoS One. 2016 May 5;11(5):e0154925. doi: 10.1371/journal.pone.0154925 (PMC4858229; doi:10.1371/journal.pone.0154925)
Supplement: S1 Table — The base positions refer to the position in the annoted NCBI L. monocytogenes EGDe reference sequence (NC_003210). The gene products are derived from the NCBI Gene database. SNP = single nucleotide polymorphism, Ins = insertion, Del = deletion, fs = frameshift. (DOC) [file pone.0154925.s002.doc]

**S1 Table. Genetic changes in the stable *L. monocytogenes* L-form. The base positions refer to the position in the annoted NCBI *L. monocytogenes* EGDe reference sequence (NC_003210). The gene products are derived from the NCBI Gene database. SNP = single nucleotide polymorphism, Ins = insertion, Del = deletion, fs = frameshift**

| **Number** | **Type** | **From** | **To** | **DNA sequence change** | **Aminoacid change** | **Gene** | **Encoded protein** |
| --- | --- | --- | --- | --- | --- | --- | --- |
| 1 | SNP | 13744 | 13744 | C --> T | Ala --> Val | lmo0010 | Mevalonate kinase |
| 2 | SNP | 13777 | 13777 | T --> C | Ile --> Thr | lmo0010 | Mevalonate kinase |
| 3 | SNP | 17197 | 17197 | C --> T | Pro --> Ser | qoxA | AA3-600 quinol oxidase subunit II |
| 4 | Ins | 35471 | 35471 | C | fs | lmo0032 | Xylose repressor |
| 5 | Del | 51560 | 51560 | T | UTR | 5' of lmo0048 | Sensor histidine kinase AgrB |
| 6 | Ins | 61308 | 61308 | A | fs | lmo0057 | Hypothetical protein |
| 7 | SNP | 62835 | 62835 | C --> T | Gln --> Stop | lmo0057 | Hypothetical protein |
| 8 | SNP | 68332 | 68332 | G --> A | Ala --> Ser | lmo0061 | Hypothetical protein |
| 9 | Ins | 81033 | 81033 | A | fs | lmo0077 | Hypothetical protein |
| 10 | Ins | 89379 | 89379 | A | fs | lmo0086 | Hypothetical protein |
| 11 | Ins | 109989 | 109989 | G | UTR | 5' of lmo0105 | Chitinase B |
| 12 | SNP | 137004 | 137004 | T --> C | UTR | 5' of lmo0135 | Peptide ABC transporter substrate-binding protein |
| 13 | Ins | 160683 | 160683 | A | fs | lmo0160 | Peptidoglycan binding protein |
| 14 | SNP | 232593 | 232593 | C --> A | UTR | 5' of sul | Dihydropteroate synthases |
| 15 | SNP | 237653 | 237654 | GA --> AG | rRNA | lmor01 | 16S ribosomal RNA |
| 16 | SNP | 237672 | 237672 | C --> T | rRNA | lmor01 | 16S ribosomal RNA |
| 17 | Ins | 247139 | 247139 | G | fs | lmor05 | 23S ribosomal RNA |
| 18 | Ins | 253089 | 253089 | T | UTR | 5' of lmo0233 | DNA repair protein RadA |
| 19 | Ins | 260154 | 260154 | G | fs | cysS | Cysteinyl-tRNA synthetase |
| 20 | Ins | 263193 | 263193 | A | fs | sigH | RNA polymerase factor sigma-70 |
| 21 | SNP | 293093 | 293093 | C --> A | Phe --> Leu | lmo0269 | Transporter |
| 22 | Ins | 312134 | 312134 | A | fs | lmo0288 | Two-component sensor histidine kinase |
| 23 | SNP | 321802 | 321802 | T --> C | Phe --> Leu | lmo0296 | Hypothetical protein |
| 24 | SNP | 335540 | 335540 | C --> T | Pro --> Leu | lmo0310 | Hypothethical protein |
| 25 | Del | 336113 | 336113 | A | fs | lmo0310 | Hypothetical protein |
| 26 | SNP | 345490 | 345490 | T --> C | Glu --> Ala | lmo0320 | Peptidoglycan-bound surface protein |
| 27 | Ins | 346666 | 346666 | A | fs | lmo0321 | Hypothetical protein |
| 28 | Ins | 348677 | 348677 | G | fs | lmo0324 | Hypothetical protein |
| 29 | Ins | 381888 | 381888 | T | UTR | 5' of lmo0354 and lmo0355 | Fatty-acid--CoA ligase and fumarate reductase subunit A |
| 30 | SNP | 401133 | 401133 | G --> A | Trp --> Stop | lmo0374 | PTS beta-glucoside transporter subunit IIB |
| 31 | Ins | 406578 | 406578 | G | UTR | 5' of lmo0382 and lmo0383 | Transcriptional regulator and methylmalonate-semialdehyde dehydrogenase |
| 32 | Ins | 416971 | 416971 | T | fs | lmo0394 | Similar to P60 protein |
| 33 | Ins | 419685 | 419685 | A | fs | lmo0398 | PTS sugar transporter subunit IIA |
| 34 | SNP | 428664 | 428664 | A --> G | Glu --> Gly | lmo0407 | Hypothetical protein |
| 35 | SNP | 441944 | 441944 | G --> A | Pro --> Leu | lmo0421 | Rod shape-determining protein RodA |
| 36 | Ins | 451201 | 451201 | C | fs | lmo0429 | Sugar hydrolase |
| 37 | Ins | 458553 | 458553 | A | fs | inlB | Internalin B |
| 38 | Ins | 472796 | 472796 | A | fs | lmo0443 | LytR family transcriptional regulator |
| 39 | Ins | 492321 | 492321 | T | fs | lmo0456 | Permease |
| 40 | Ins | 501891 | 501891 | A | fs | lmo0464 | Transposase |
| 41 | SNP | 550886 | 550886 | T --> C | Gln --> Arg | lmo0516 | Encapsulation protein CapA |
| 42 | Ins | 563927 | 563927 | T | fs | lmo0527 | Transmembrane protein |
| 43 | Ins | 596536 | 596536 | A | fs | lmo0557 | Phosphoglycerate mutase |
| 44 | Ins | 623840 | 623840 | A | fs | lmo0584 | Hypothetical protein |
| 45 | Ins | 634287 | 634287 | T | UTR | 5' of lmo0593 | Formate transporter |
| 46 | Del | 668832 | 668843 | ACACCAAAAGAT |  | lmo0627 | Peptidoglycan bound protein |
| 47 | Ins | 705507 | 705507 | A | fs | lmo0668 | ABC transporter permease |
| 48 | Ins | 717498 | 717498 | A | fs | lmo0681 | Flagellar biosynthesis regulator FlhF |
| 49 | SNP | 756709 | 756709 | C --> T | UTR | 5' of lmo0727 | Glucosamine--fructose-6-phosphate aminotransferase |
| 50 | SNP | 765571 | 765571 | G --> A | UTR | 5' of lmo0734 | LacI family transcriptional regulator |
| 51 | Ins | 784889 | 784889 | A | fs | lmo0759 | Hypothetical protein |
| 52 | Del | 797443 | 797443 | T | fs | lmo0771 | Hypothetical protein |
| 53 | Del | 811214 | 811214 | A | fs | lmo0786 | ACP phosphodiesterase |
| 54 | Ins | 841287 | 841287 | G | fs | lmo0814 | Oxidoreductase |
| 55 | Ins | 841446 | 841446 | A | fs | lmo0814 | Oxidoreductase |
| 56 | Del | 843143 | 843143 | A | fs | lmo0816 | Regulatory protein PaiA |
| 57 | SNP | 855339 | 855339 | T --> C | Cys --> Arg | lmo0828 | Transposase |
| 58 | SNP | 899282 | 899282 | A --> G | Thr --> Ala | lmo0860 | Sugar ABC transporter permease |
| 59 | Ins | 920466 | 920466 | T | fs | lmo0881 | Hypothetical protein |
| 60 | Ins | 933371 | 933371 | G | fs | lmo0897 | Transporter |
| 61 | SNP | 943887 | 943887 | A --> G | Gln --> Arg | lmo0907 | Phosphoglycerate mutase |
| 62 | SNP | 964343 | 964343 | C --> T | Gly --> Arg | lmo0927 | Hypothetical protein |
| 63 | Ins | 979569 | 979569 | T | UTR | 5' of lmo0944 | Hypothetical protein |
| 64 | Del | 996629 | 996629 | A | fs | lmo0963 | Heat shock protein HtpX |
| 65 | Ins | 1029971 | 1029971 | A | fs | lmo0999 | Hypothetical protein |
| 66 | Ins | 1030835 | 1030835 | C | fs | lmo1000 | Phytoene dehydrogenase |
| 67 | Del | 1053212 | 1053212 | T | fs | lmo1024 | Hypothetical protein |
| 68 | Ins | 1057300 | 1057300 | T | fs | 5' of lmo1028 | Hypothetical protein |
| 69 | SNP | 1072102 | 1072102 | T --> C | Leu --> Pro | lmo1042 | Molybdopterin biosynthesis protein MoeA |
| 70 | Ins | 1088017 | 1088017 | A | fs | lmo1061 | Two-component sensor histidine kinase |
| 71 | Ins | 1088643 | 1088643 | T | fs | lmo1061 | Two-component sensor histidine kinase |
| 72 | SNP | 1093655 | 1093655 | A --> G | Glu --> Gly | lmo1066 | Myo-inositol-1(or 4)-monophosphatase |
| 73 | SNP | 1120429 | 1120429 | T --> C | Trp --> Arg | lmo1085 | Teichoic acid biosynthesis protein B |
| 74 | Del | 1171241 | 1171285 | GTAGATCCGGTAGATCCGGTAGATCCGGTAGATCCGGTAGATCCA |  | lmo1138 | ATP-dependent Clp protease proteolytic subunit |
| 75 | Ins | 1179772 | 1179772 | T | UTR |  |  |
| 76 | Ins | 1180082 | 1180082 | T | UTR |  |  |
| 77 | SNP | 1200147 | 1200147 | G --> A | Val --> Ile | lmo1172 | Two-component response regulator |
| 78 | SNP | 1209680 | 1209680 | G --> A | Ala --> Thr | lmo1181 | Cobalamin adenosyl transferase |
| 79 | Ins | 1229619 | 1229619 | T | fs | cbiQ | Cobalamin biosynthesis protein CbiQ |
| 80 | Ins | 1251192 | 1251192 | A | fs | lmo1226 | Transporter |
| 81 | Del | 1257096 | 1257096 | A | fs | lmo1232 | Recombination and DNA strand exchange inhibitor protein |
| 82 | Del | 1262002 | 1262002 | A | fs | lmo1235 | Aspartate kinase |
| 83 | Del | 1263790 | 1263790 | A | UTR | 5' of lmo1236 and racE | Hypothetical protein and glutamate racemase |
| 84 | SNP | 1270587 | 1270587 | C --> T | Glu --> Lys | lmo1245 | Hypothetical protein |
| 85 | Ins | 1271991 | 1271991 | A | fs | lmo1246 | ATP-dependent RNA helicase |
| 86 | Del | 1276715 | 1276715 | A | UTR | 5' of lmo1252 | Hypothetical protein |
| 87 | SNP | 1280815 | 1280815 | T --> C | Asn --> Asp | lmo1255 | PTS trehalose transporter subunit IIBC |
| 88 | Del | 1326407 | 1326407 | A | UTR | 5' of glnR | Glutamine synthetase repressor |
| 89 | Ins | 1359324 | 1359324 | T | UTR | 5' of pnpA | Polynucleotide phosphorylase |
| 90 | Ins | 1364017 | 1364017 | G | UTR | 5' of lmo1336 | 5-formyltetrahydrofolate cyclo-ligase |
| 91 | Ins | 1371863 | 1371863 | G | fs | comGA | Competence protein ComGA |
| 92 | Del | 1422193 | 1422193 | A | UTR | 5' of lmo1395 | Hypothetical protein |
| 93 | Ins | 1425180 | 1425180 | A | UTR | 5' of recA | Recombinase A |
| 94 | Ins | 1440766 | 1440766 | A | fs | lmo1410 | Hypothetical protein |
| 95 | SNP | 1451383 | 1451383 | C --> T | Gly --> Glu | murB | UDP-N-acetylenolpyruvoylglucosamine reductase |
| 96 | SNP | 1452839 | 1452839 | T --> C | Leu --> Pro | lmo1422 | Glycine/betaine ABC transporter permease |
| 97 | Ins | 1460610 | 1460610 | G | fs | lmo1429 | Hypothetical protein |
| 98 | SNP | 1472045 | 1472045 | C --> T | Gly --> Asp | lmo1438 | Penicillin-binding protein |
| 99 | Ins | 1499179 | 1499179 | G | fs | lmo1466 | Hypothetical protein |
| 100 | SNP | 1508114 | 1508114 | G --> A | Pro --> Leu | hrcA | Heat-inducible transcription repressor |
| 101 | Ins | 1521504 | 1521504 | A | fs | lmo1489 | Hypothetical protein |
| 102 | Ins | 1528106 | 1528106 | T | fs | udk | Uridine kinase |
| 103 | SNP | 1535675 | 1535675 | C --> T | Ala --> Thr | lmo1505 | ABC transporter ATP-binding protein |
| 104 | Ins | 1543873 | 1543873 | A | fs | 5' of lmo1511 | Hypothetical protein |
| 105 | Ins | 1548247 | 1548247 | A | fs | lmo1515 | Hypothetical protein |
| 106 | Ins | 1553201 | 1553201 | A | fs | hisS | Histidyl-tRNA synthetase |
| 107 | Del | 1582674 | 1582674 | T | fs | mreB | Rod shape-determining protein MreB |
| 108 | Ins | 1583804 | 1583804 | T | fs | radC | DNA repair protein RadC |
| 109 | SNP | 1597251 | 1597251 | A --> G | UTR | 5' of thrS | Threonyl-tRNA synthetase |
| 110 | Ins | 1633344 | 1633344 | T | fs | argC | N-acetyl-gamma-glutamyl-phosphate reductase |
| 111 | Del | 1642868 | 1642868 | T | fs | ccpA | Catabolite control protein A |
| 112 | Ins | 1646242 | 1646242 | T | fs | lmo1602 | Hypothetical protein |
| 113 | SNP | 1658476 | 1658476 | T --> C | Asp --> Gly | lmo1616 | Hypothetical protein |
| 114 | Ins | 1659147 | 1659147 | A | fs | lmo1616 | Hypothetical protein |
| 115 | Ins | 1672857 | 1672857 | T | fs | trpC | Indole-3-glycerol phosphate synthase |
| 116 | SNP | 1688915 | 1688915 | A --> G | Trp --> Arg | lmo1644 | Helicase SNF2 |
| 117 | SNP | 1708957 | 1708957 | A --> T | Ile --> Asn | leuS | Leucyl-tRNA synthetase |
| 118 | SNP | 1712381 | 1712381 | T --> C | Val --> Ala | lmo1661 | Hypothetical protein |
| 119 | Del | 1758493 | 1758493 | A | fs | lmo1694 | CDP-abequose synthase |
| 120 | SNP | 1791276 | 1791276 | C --> T | Gly --> Arg | lmo1728 | Cellobiose phosphorylase |
| 121 | SNP | 1795076 | 1795076 | C --> A | Val --> Leu | lmo1729 | Beta-glucosidase |
| 122 | SNP | 1796399 | 1796399 | C --> T | Ser --> Phe | lmo1730 | Sugar ABC transporter substrate-binding protein |
| 123 | Ins | 1816426 | 1816426 | A | fs | lmo1746 | ABC transporter permease |
| 124 | Ins | 1816882 | 1816882 | A | fs | lmo1746 | ABC transporter permease |
| 125 | Ins | 1854543 | 1854543 | C | fs | lmo1777 | Hypothetical protein |
| 126 | Del | 1870244 | 1870267 | GCATCCGCATCGGCATCCGCGTCA |  | lmo1799 | Peptidoglycan binding protein |
| 127 | Del | 1870292 | 1870333 | GCGTCAGCATCCGCATCCGCATCAGCATCCGCGTCGGCATCC |  | lmo1799 | Peptidoglycan binding protein |
| 128 | Del | 1870346 | 1870351 | GCATCC |  | lmo1799 | Peptidoglycan binding protein |
| 129 | Del | 1871006 | 1871011 | GCATCA |  | lmo1799 | Peptidoglycan binding protein |
| 130 | Del | 1871414 | 1871425 | GCGTCAGCATCC |  | lmo1799 | Peptidoglycan binding protein |
| 131 | Ins | 1879415 | 1879415 | T | fs | smc | Chromosome condensation protein Smc |
| 132 | SNP | 1883196 | 1883196 | G --> A | His--> Tyr | plsX | Glycerol-3-phosphate acyltransferase PlsX |
| 133 | Ins | 1921300 | 1921300 | C | fs | lmo1845 | Hypothetical protein |
| 134 | Ins | 1938806 | 1938806 | A | fs | lmo1865 | Hypothetical protein |
| 135 | Ins | 1950499 | 1950499 | A | fs | lmo1875 | ABC transporter ATP-binding protein |
| 136 | Ins | 1995206 | 1995206 | T | fs | lmo1919 | Hypothetical protein |
| 137 | SNP | 2027034 | 2027034 | A --> G | Leu --> Ser | lysA | L-alanoyl-D-glutamate peptidase |
| 138 | SNP | 2032865 | 2032865 | C --> T | Trp --> Stop | fhuB | Ferrichrome ABC transporter permease |
| 139 | SNP | 2041850 | 2041850 | C --> T | Arg --> Ser | lmo1967 | Toxic ion resistance protein |
| 140 | Ins | 2045965 | 2045965 | T | fs | lmo1972 | PTS pentitol transporter subunit IIB |
| 141 | Ins | 2054132 | 2054132 | T | UTR | 5' of lmo1982 and ilvD | Hypothetical protein and dihydroxy-acid dehydratase |
| 142 | SNP | 2057808 | 2057808 | G --> A | Arg --> Lys | ilvB | Acetolactate synthase |
| 143 | Ins | 2070642 | 2070642 | T | fs | lmo1996 | DeoR family transcriptional regulator |
| 144 | Ins | 2081553 | 2081553 | A | fs | lmo2008 | ABC transporter permease |
| 145 | Del | 2106003 | 2106003 | A | UTR |  |  |
| 146 | Ins | 2115251 | 2115251 | T | UTR | 5' of ftsA | Cell division protein ftsA |
| 147 | Del | 2119848 | 2119848 | T | fs | murE | UDP-N-acetylmuramoylalanyl-D-glutamate--2,6-diaminopimelate ligase |
| 148 | SNP | 2124511 | 2124511 | T --> C | Glu --> Gly | mraW | S-adenosyl-methyltransferase MraW |
| 149 | Ins | 2125647 | 2125647 | A | UTR | 5' of lmo2042 | Cell division protein MraZ |
| 150 | Ins | 2132844 | 2132844 | A | fs | lmo2050 | Excinuclease ABC subunit A |
| 151 | Ins | 2134577 | 2134577 | G | fs | lmo2050 | Excinuclease ABC subunit A |
| 152 | Ins | 2135129 | 2135129 | T | fs | lmo2051 | Hypothetical protein |
| 153 | Ins | 2140048 | 2140048 | A | fs | ctaA | Heme O oxygenase |
| 154 | Ins | 2143952 | 2143952 | C | fs | lmo2062 | Copper transporter |
| 155 | Ins | 2146406 | 2146406 | A | UTR | 5' of lmo2066 | Hypothetical protein |
| 156 | Ins | 2147513 | 2147513 | A | UTR | 5' of lmo2067 | Bile acid hydrolase |
| 157 | Ins | 2150099 | 2150099 | A | fs | lmo2070 | Hypothetical protein |
| 158 | SNP | 2163425 | 2163425 | T --> A | Asn --> Ile | lmo2085 | Peptidoglycan binding protein |
| 159 | Del | 2190025 | 2190025 | A | fs | lmo2109 | Hydrolase |
| 160 | Ins | 2193664 | 2193664 | A | fs | lmo2114 | ABC transporter ATP-binding protein |
| 161 | SNP | 2201334 | 2201334 | T --> C | Thr --> Ala | lmo2120 | Hypothetical protein |
| 162 | Del | 2215329 | 2215329 | T | UTR | 5' of lmo2130 and lmot41 | Hypothetical protein and tRNA |
| 163 | Ins | 2224402 | 2224402 | T | fs | lmo2140 | ABC transporter permease |
| 164 | Del | 2233836 | 2233836 | T | UTR |  |  |
| 165 | SNP | 2238069 | 2238069 | A --> G | Met --> Thr | lmo2155 | Ribonucleotide-diphosphate reductase subunit alpha |
| 166 | Ins | 2252024 | 2252024 | T | fs | lmo2171 | MFS transporter |
| 167 | Ins | 2257756 | 2257756 | G | fs | fabG | 3-ketoacyl-ACP reductase |
| 168 | Ins | 2259715 | 2259715 | A | UTR |  |  |
| 169 | Ins | 2265223 | 2265223 | T | fs | lmo2179 | Peptidoglycan binding protein |
| 170 | SNP | 2281918 | 2281918 | T --> C | His --> Arg | lmo2193 | Peptide ABC transporter ATP-binding protein |
| 171 | Del | 2288555 | 2288555 | T | UTR |  |  |
| 172 | SNP | 2300629 | 2300629 | A --> G | Val --> Ala | hemH | Ferrochelatase |
| 173 | Del | 2337616 | 2337616 | A | UTR | 5' of lmo2247 and lmo2248 | Oxidoreductase and hypothetical protein |
| 174 | SNP | 2339072 | 2339072 | T --> C | Leu --> Ser | lmo2249 | Low-affinity inorganic phosphate transporter |
| 175 | Del | 2347425 | 2347425 | T | fs | lmo2260 | Hypothetical protein |
| 176 | Missing Prophage A118 | 2360623 | 2402410 |  |  | lmo2270-lmo2333 |  |
| 177 | SNP | 2411854 | 2411854 | T --> C | UTR | 5' of lmo2342 | 16S pseudouridylate synthase |
| 178 | SNP | 2415937 | 2415937 | G --> A | Pro --> Leu | lmo2347 | Amino acid ABC transporter permease |
| 179 | Del | 2425586 | 2425586 | A | UTR | 5' of lmo2356 | Hypothetical protein |
| 180 | Del | 2431374 | 2431374 | T | UTR | 5' of lmo2361 and lmo2362 | Hypothetical protein and amino acid antiporter |
| 181 | Del | 2436266 | 2436266 | T | UTR | 5' of lmo2365 | RofA family transcriptional regulator |
| 182 | SNP | 2443922 | 2443922 | T --> C | Lys --> Glu | lmo2366 | DeoR family transcriptional regulator |
| 183 | Ins | 2445538 | 2445538 | A | fs | pgi | Glucose-6-phosphate isomerase |
| 184 | Ins | 2461585 | 2461585 | A | fs | lmo2386 | Hypothetical protein |
| 185 | Ins | 2463043 | 2463043 | C | UTR |  |  |
| 186 | Ins | 2510165 | 2510165 | C | UTR | 5' of lmo2443 | Hypothetical protein |
| 187 | SNP | 2585360 | 2585360 | A --> G | Val --> Ala | lmo2508 | Hypothetical protein |
| 188 | SNP | 2594027 | 2594027 | G --> T | Thr --> Lys | lmo2515 | Two-component response regulator DegU |
| 189 | Del | 2598855 | 2598855 | T | fs | lmo2520 | O-succinylbenzoate-CoA synthase |
| 190 | Ins | 2601410 | 2601410 | T | fs | lmo2523 | Single-strand DNA-binding protein |
| 191 | SNP | 2605829 | 2605829 | A --> T | Asp --> Glu | atpC | ATP synthase F0F1 subunit epsilon |
| 192 | SNP | 2639884 | 2639884 | A --> G | UTR | 5' of pyrG | CTP synthase |
| 193 | Ins | 2643773 | 2643773 | T | fs | lmo2564 | 4-oxalocrotonate isomerase |
| 194 | Ins | 2644164 | 2644164 | T | fs | lmo2565 | Hypothetical protein |
| 195 | SNP | 2652588 | 2652588 | A --> T | Asp --> Glu | lmo2575 | Cation transporter |
| 196 | Ins | 2653974 | 2653974 | T | fs | lmo2576 | Colossin A |
| 197 | Ins | 2678937 | 2678937 | T | fs | lmo2590 | ATP-binding protein |
| 198 | SNP | 2687553 | 2687553 | C --> T | Ala --> Thr | cbiO | Cobalt ABC transporter ATP-binding subunit |
| 199 | Ins | 2723582 | 2723582 | A | fs | lmo2651 | PTS mannitol transporter subunit IIA |
| 200 | SNP | 2758122 | 2758122 | T --> C | Val --> Ala | lmo2684 | PTS cellbiose transporter subunit IIC |
| 201 | Del | 2786408 | 2786408 | A | UTR | 5' of lmo2712 | Gluconate kinase |
| 202 | Ins | 2841385 | 2841385 | G | fs | lmo2760 | ABC transporter ATP-binding protein |
| 203 | Ins | 2869338 | 2869338 | A | fs | lmo2784 | Transcriptional antiterminator |
| 204 | SNP | 2873338 | 2873338 | C --> T | Trp --> Stop | bvrC | ADP-ribosylglycohydrolase |
| 205 | Ins | 2903098 | 2903098 | A | fs | lmo2818 | MFS transporter |
| 206 | Ins | 2936440 | 2936440 | A | fs | lmo2848 | L-rhamnose isomerase |
