# Supplementary material for: The Absence of a Mature Cell Wall Sacculus in Stable Listeria monocytogenes L-Form Cells Is Independent of Peptidoglycan Synthesis
Source: PLoS One. 2016 May 5;11(5):e0154925. doi: 10.1371/journal.pone.0154925 (PMC4858229; doi:10.1371/journal.pone.0154925)
Supplement: S2 Table — Prha = rhamnose inducible promoter, Phelp = highly expressed, constitutive promoter. (DOC) [file pone.0154925.s003.doc]

**S2 Table. The stable *L. monocytogenes* L-form was transformed with the following combinations of plasmids in order to test for reversion to the rod shape.** P_rha_ = rhamnose inducible promoter [1], P_help_ = highly expressed, constitutive promoter [2]

| **Plasmid 1** | **Plasmid 2** |
| --- | --- |
| pIMK3/*lmo0421* [3] | - |
| pIMK3/*lmo1438* | - |
| pLEB579/P_rha_-*mreB* [4] | - |
| pIMK3/*lmo1438* | pLEB579/P_help_-*lmo0421* |
| pIMK3/*lmo0421* | pLEB579/P_rha_-*mreB* |
| pIMK3/*lmo1438* | pLEB579/P_rha_-*mreB* |
| pIMK3/*lmo1438* | pLEB579/P_rha_-*mreB*-P_help_-*lmo0421* |

**References**

1. Fieseler L, Schmitter S, Teiserskas J, Loessner MJ. Rhamnose-inducible gene expression in *Listeria monocytogenes*. Plos One. 2012;7(8):e43444.

2. Riedel CU, Monk IR, Casey PG, Morrissey D, O'Sullivan GC, Tangney M, et al. Improved luciferase tagging system for *Listeria monocytogenes* allows real-time monitoring *in vivo* and *in vitro*. Appl Environ Microbiol. 2007;73(9):3091-4.

3. Monk IR, Gahan CG, Hill C. Tools for functional postgenomic analysis of *Listeria monocytogenes*. Appl Environ Microbiol. 2008;74(13):3921-34.

4. Beasley SS, Takala TM, Reunanen J, Apajalahti J, Saris PE. Characterization and electrotransformation of *Lactobacillus crispatus* isolated from chicken crop and intestine. Poultry science. 2004;83(1):45-8.
